# Spatial Heterogeneity of Leaf Area Index (LAI) and Its Temporal Course on Arable Land: Combining Field Measurements, Remote Sensing and Simulation in a Comprehensive Data Analysis Approach (CDAA)

**DOI:** 10.1371/journal.pone.0158451

**Published:** 2016-07-08

**Authors:** Tim G. Reichenau, Wolfgang Korres, Carsten Montzka, Peter Fiener, Florian Wilken, Anja Stadler, Guido Waldhoff, Karl Schneider

**Affiliations:** 1 Institute of Geography, University of Cologne, Cologne, Germany; 2 Institute of Bio- and Geosphere: Agrosphere (IBG 3), Research Centre Jülich, Jülich, Germany; 3 Institut für Geographie, Universität Augsburg, Augsburg, Germany; 4 Geopedology and Landscape Development, Brandenburg University of Technology Cottbus–Senftenberg, Cottbus, Germany; 5 Institute of Soil Landscape Research, Leibniz-Centre for Agricultural Landscape Research (ZALF) e.V., Müncheberg, Germany; 6 Institute of Crop Science and Resource Conservation, University of Bonn, Bonn, Germany; Tennessee State University, UNITED STATES

## Abstract

The ratio of leaf area to ground area (leaf area index, LAI) is an important state variable in ecosystem studies since it influences fluxes of matter and energy between the land surface and the atmosphere. As a basis for generating temporally continuous and spatially distributed datasets of LAI, the current study contributes an analysis of its spatial variability and spatial structure. Soil-vegetation-atmosphere fluxes of water, carbon and energy are nonlinearly related to LAI. Therefore, its spatial heterogeneity, i.e., the combination of spatial variability and structure, has an effect on simulations of these fluxes. To assess LAI spatial heterogeneity, we apply a Comprehensive Data Analysis Approach that combines data from remote sensing (5 m resolution) and simulation (150 m resolution) with field measurements and a detailed land use map. Test area is the arable land in the fertile loess plain of the Rur catchment on the Germany-Belgium-Netherlands border. LAI from remote sensing and simulation compares well with field measurements. Based on the simulation results, we describe characteristic crop-specific temporal patterns of LAI spatial variability. By means of these patterns, we explain the complex multimodal frequency distributions of LAI in the remote sensing data. In the test area, variability between agricultural fields is higher than within fields. Therefore, spatial resolutions less than the 5 m of the remote sensing scenes are sufficient to infer LAI spatial variability. Frequency distributions from the simulation agree better with the multimodal distributions from remote sensing than normal distributions do. The spatial structure of LAI in the test area is dominated by a short distance referring to field sizes. Longer distances that refer to soil and weather can only be derived from remote sensing data. Therefore, simulations alone are not sufficient to characterize LAI spatial structure. It can be concluded that a comprehensive picture of LAI spatial heterogeneity and its temporal course can contribute to the development of an approach to create spatially distributed and temporally continuous datasets of LAI.

## Introduction

Fluxes of matter and energy are very heterogeneous in space and time [1] and references therein. In terrestrial ecosystems, the exchange-processes of water, carbon, and energy between soil and atmosphere on vegetated surfaces are largely influenced by plants. The rates of these exchange processes (fluxes) as well as the state variables are a result of interaction of biotic and abiotic factors like vegetation properties, topography, soil properties and weather [2]. Particularly vegetation type and weather are of key importance to understand the vegetation control upon energy and matter cycles [3–5].

Many studies address issues of spatial heterogeneity of fluxes and vegetation state variables in near natural ecosystems (e.g. [6, 7]). However, in many parts of the world arable land occupies large areas (24% of the EU-28) [8]. On arable land, in addition to the heterogeneity caused by environmental aspects such as soil texture [9], there are direct anthropogenic impacts. These are on the one hand the shape, size, and position of arable fields and, on the other hand, agricultural management decisions like choice of crops and timing, type and intensity of cultivation, and fertilization activities [10].

Leaf area index (LAI, one-sided green leaf area per unit ground surface area) is a key variable determining the exchange of matter and energy between the surface and the atmosphere [2]. Among others, LAI largely controls transpiration and thus the partitioning of infiltrated water into evapotranspiration and percolation. It governs the interception of precipitation and determines the absorption of light for photosynthesis. Therefore, LAI is also an important driving variable for many ecological models [11, 12]. Furthermore, it has been shown that the temporal variability of LAI can have a significant impact on soil moisture simulations [13].

Due to nonlinearities, flux calculations based on a spatially uniform value of LAI differ from those considering LAI spatial heterogeneity. Especially at the beginning and the end of the growing season, LAI of arable land changes quickly. Thus, addressing the temporal development of LAI along with its spatial heterogeneity is of major importance in determining matter and energy balances on the catchment or regional scale.

### Assessing LAI and its spatial heterogeneity

A number of methods have been developed to analyze LAI spatial heterogeneity on different scales. An overview about different methods is provided by [14]. These methods differ in precision, coverage and spatial as well as temporal resolution.

#### Field measurements

The reference method to quantify actual LAI is destructive measurement. Plant material is sampled by harvesting leaves and measuring the leaf area using a leaf area meter or scanner. This procedure is rather time consuming and labor intensive. Thus, it can usually only be applied to a limited number of locations and dates [14–16]. This typically limits the method to small areas and therefore usually does not allow the analysis of spatial structure.

#### Remote sensing

Many remote sensing methods are based on empirical relations between the measured variable and LAI. Fine spatial resolution can be achieved by using terrestrial laser scanning together with a model to derive LAI from plant height [17, 18]. Many airborne and spaceborne remote sensing approaches utilize concepts like the Normalized Difference Vegetation Index (NDVI) or other vegetation indices; others use radiation transfer models [19]. Their spatial resolution ranges from centimeters (HyPlant) [20] over meters (IKONOS, e.g. [21]) to a kilometer (e.g. AVHRR, [22]). The spatial extent of remote sensing datasets ranges from single fields (small unmanned airborne vehicles, e.g. [23]) to several km^2^ (airborne sensors on aircraft, e.g. CASI, [24, 25]) up to continents or the globe (spaceborne, e.g. MODIS). Spatial coverage of airborne and spaceborne data can be limited due to cloud cover (optical systems). Temporal availability is usually inversely proportional to spatial resolution and extent and may be increased by using multiple platforms in orbit.

The nonlinear relation between vegetation indices and LAI saturates when additional leaves do not have an effect on reflectance. This occurs at LAI values between 2 and 5, depending on the index [24]. For NDVI, relations have been reported to saturate at LAI values of 2 to 4 [26, 27], but Asrar et al. [28] report saturation at 6 for wheat.

Hemispherical photography is frequently used in forest ecosystems [29] but also for crops [30]. This method uses pictures obtained with a fisheye lens. Plant area is estimated based on the identification of background (soil or sky) and plant (green) pixels and on geometric considerations. However, in order to obtain the LAI, the plant area index derived from hemispherical photography must be corrected appropriately.

### Simulation

Spatially and temporally continuous estimates of LAI and its heterogeneity can be generated using spatially explicit models. Current process-oriented, physically based models are able to reproduce LAI measurements on the point scale with appropriate quality if adequate data on weather, soil and crop properties are available (e.g. [31, 32]). The quality of model results strongly depends on the input data. For models of large areas like catchments, weather data are often interpolated from station measurements and soil properties are estimated from maps. Therefore, these models often produce larger errors as compared to well-controlled simulations in individual field studies. Thus, since the heterogeneity of the input data provided to the models is typically smaller than the respective heterogeneities in the field, the resulting variability of simulated LAI is assumed to be smaller than the observed variability in the field.

### The current study

#### Comprehensive Data Analysis Approach

Our Comprehensive Data Analysis Approach (CDAA) combines data from several sources. Datasets with different temporal and spatial resolutions and different degrees of uncertainty complement each other and compensate disadvantages of single-source datasets. In the current study, data from field measurements, spaceborne remote sensing and simulation are analyzed. While field measurements provide the reference on the point scale, remote sensing provides information on the measured spatial distribution of LAI. Simulation results complement the analysis by providing a continuous temporal course of LAI development. A detailed land use dataset allows the discrimination of crops in the remote sensing data and thus enables a differentiated analysis and explanation of the complex patterns of LAI heterogeneity. Our CDAA enables a comprehensive analysis of the temporal course of LAI spatial heterogeneity. Inhomogeneities, inconsistencies or breaks may be used to identify errors or limitations of the respective datasets such as misclassifications in the land use map, saturation effects in remote sensing data or parameterization issues in the model.

#### Aims and hypotheses

The aims of this study are (i) to compile a consistent, regional LAI dataset from field measurements, remote sensing data, and simulation results and (ii) to use this dataset to analyze, explain, and quantify the spatial heterogeneity and structure of LAI and its temporal development. This is accomplished using the CDAA.

The underlying hypotheses are:

H1 The temporal course of spatial variability of LAI estimated by remote sensing and simulation compares reasonably well with field measurements.
H2 The CDAA (Comprehensive Data Analysis Approach) provides a comprehensive picture of LAI spatial heterogeneity and its temporal development.

## Materials and Methods

### Test area

**Fig 1:**
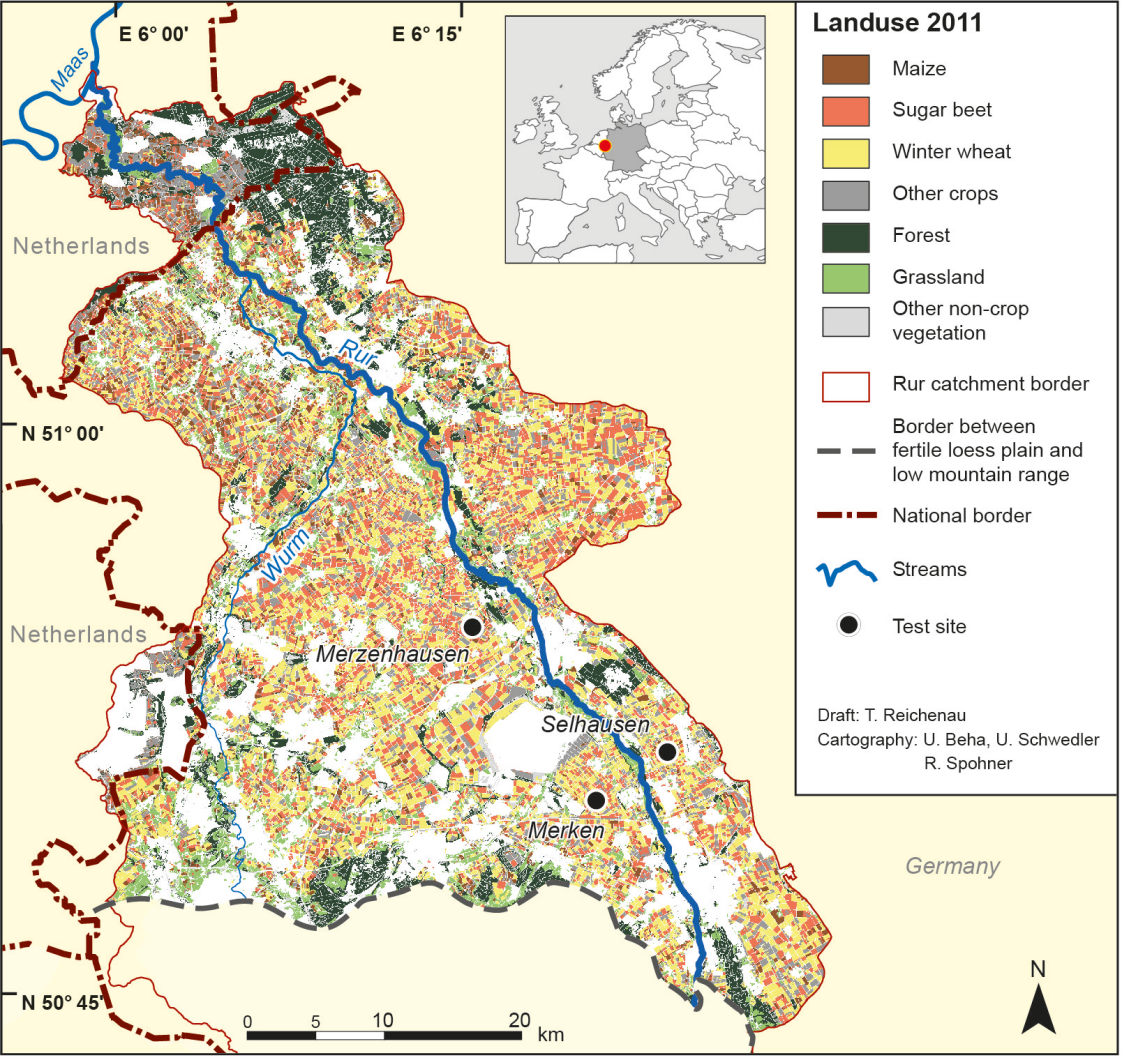
Map of the test area. Land use map (2011) and location of field measurement sites for the fertile loess plain in the northern part of the Rur catchment. White areas are non-vegetated.

The study was carried out for the northern part of the Rur catchment, a 1100 km^2^ area located at the German-Netherlands border with 100 km^2^ belonging to the Netherlands (see Fig 1). The fertile loess plain is located between the low mountain range of the Eifel in the south and the confluence of the rivers Rur and Maas in the north. Slopes are less than 4° in the test area. The land use is 47% arable land. The main crops are winter wheat (41% of the arable area), sugar beet (28%) and maize (10%). The fertile loess plain has a mean elevation of about 100 m above sea level. The mid-latitude, warm temperate climate has an annual precipitation of about 700 mm and a mean annual air temperature of about 10°C. The major soils are Haplic Luvisols and Cumulic Anthrosols near the drainage lines, both with silt loam textures. Soils with a loamy sand texture (Fimic Anthrosols and Dystric Cambisols) are located on the northern edge of the loess plain. Soils close to the river Rur are Gleysols and Fluvisols with silty loam and loamy sand textures. The test area is the research area of the Transregional Collaborative Research Center 32 (TR32) “Patterns in Soil-Vegetation-Atmosphere-Systems: Monitoring, Modelling and Data Assimilation” [33, 34]. It is also part of the Terrestrial Environmental Observatories (TERENO) infrastructure [35].

#### Land use dataset

The 15 m resolution land use classification of 2011 [36] (DOI:10.5880/TR32DB.7) for the Rur catchment was generated by using the Multi-Data Approach (MDA) [37]. The MDA combines supervised classification results of multi temporal moderate spatial resolution remote sensing data (e.g. Landsat) with additional geodata sets like ATKIS (Authorative Topographic-Cartographic Information System) to enhance the information content of the resulting land use data. For the improved differentiation of agricultural crops in the land use classification of 2011, the phenology of the major crop types was investigated. On this basis, remote sensing scenes of specific periods during the growing season were analyzed, where individual crop types could be distinguished most favorably based on their spectral appearance [38]. The final crop classification is based on RapidEye remote sensing data from 05/21, 05/30, 06/27 and 08/02. Full spatial coverage was not available on all dates. To further enhance the crop differentiation, annually updated Physical Block data were incorporated to delimit the arable land in the test area [39]. Individual Physical Blocks delimit coherent land parcels of the same principle agricultural land use type (i.e. arable land, permanent crops or grassland) on a scale of 1:10.000. They were used to reduce misclassifications with other vegetation types. The land use classification has an overall accuracy of 85.97%. For the current study, it was upscaled to lower resolutions using the majority approach by assigning the most frequent land use class.

### Field measurements (*field*)

Destructive measurements of LAI for winter wheat, maize and sugar beet were carried out about biweekly on fields located in Selhausen (50°52’00”N 6°27’01”E), Merken (5°50’47”N 6°24’04”E) and Merzenhausen (50°55’47”N 6°17’46”E) (Fig 1) in the years 2008 until 2012 in the context of field campaigns to monitor vegetation and soil moisture [32, 40]. Field samples were taken with permission of the respective farmers. Three to twelve points per field were sampled on six to 13 dates during the growing season (Table 1). To determine LAI two equivalent approaches were used:

1. One running meter of plant material was cut at each sampling point. Plants were separated into the different plant organs. The LI-3100C (LI-COR Biosciences Lincoln, Nebraska, USA) area meter was used in the lab to determine the leaf area [14–16]. The device was calibrated according to the device’s operation manual at the beginning of the growing season. Dividing measured leaf area by the row spacing (wheat) or by the number of plants per m^2^ (sugar beet) resulted in the respective LAI.
2. Either 0.4 or 0.5 running meters from three rows of plants (wheat) or three plants (sugar beet, maize) were taken from the field. Leaf area of an aliquot of the plant material was measured using a LI-3000A Portable Area Meter with LI-3050 Transparent Belt Conveyor Accessory (LI-COR Biosciences Lincoln, Nebraska, USA) in the lab. Measured leaf area was corrected based on calibration plates with known area. Upscaling to the square meter was done using the area to fresh weight ratio and the number of plants per square meter (based on counting three two-meter rows and row distance in the field).

**Table 1:** Dates, sites and methods for the destructive field measurements of LAI. Descriptions of the methods 1 and 2 are given in the text.

crop	year	site	period of measurement	number of dates	number of points	method
winter wheat	2008	Selhausen	08/03/04–08/07/24	10	3	2
sugar beet	2008	Selhausen	08/06/10–08/09/17	8	3	2
maize	2008	Selhausen	08/06/17–08/09/17	8	3	2
winter wheat	2009	Merken	09/03/26–08/07/27	10	3	2
winter wheat	2009	Selhausen	08/12/10–09/07/16	13	3	2
sugar beet	2009	Selhausen	09/05/18–09/10/06	11	3	2
sugar beet	2009	Merken	09/05/18–09/09/27	10	3	2
maize	2009	Selhausen	09/04/16–09/08/08	8	3	2
winter wheat	2010	Selhausen	10/04/01–10/07/22	9	6	2
sugar beet	2010	Selhausen	10/05/27–10/10/28	12	7	2
winter wheat	2011	Merzenhausen	11/04/18–11/08/01	10	12	2
winter wheat	2011	Selhausen	11/03/23–11/07/19	9	8	1
winter wheat	2012	Merzenhausen	12/04/05–12/07/25	12	12	2
winter wheat	2012	Selhausen	12/03/30–12/07/24	9	8	1
sugar beet	2012	Selhausen	12/06/09–12/09/03	6	8	1

Irregular values due to measurement error [15] were eliminated by comparison with LAI values measured at other sampling points on the same date and in the same field, and by analyzing the temporal course within the growing season.

In this study, the field measurement dataset is called “*field*” (field measurements, DOI: 10.5880/TR32DB.20).

### Spaceborne remote sensing

With a constellation of five identical satellites, RapidEye provides the potential for daily coverage of multispectral imagery with a 6.5 m spatial resolution. In this study, the Level 3A product was used, which is already radiometrically and geometrically corrected, resampled to 5 m spatial resolution and delivered in 25*25 km tiles. In order to cover the whole test area, five tiles of seven cloud-free dates in 2011 (02/22, 03/03, 04/02, 04/07, 06/27, 09/25, 10/22) were mosaicked.

For deriving biophysical variables like LAI from spaceborne multispectral remote sensing data, typically an empirical relationship to a vegetation index is used [41]. Here, the Normalized Difference Vegetation Index (NDVI) was used, which is mainly sensitive to leaf chlorophyll content and is calculated as follows:

$$\text{NDVI}=\frac{\text{NIR}\;-\;\text{Red}}{\text{NIR}\;+\;\text{Red}}$$ (1)

where NIR is the near-infrared band value (760–850 nm) and Red is the red band value (630–685 nm). Following the MODIS LAI product generation approach [42, 43], the derived NDVI map was then used to estimate Fractional Vegetation Cover (FVC) as follows [44, 45]:

$$\text{FVC}=\frac{\text{NDVI}-{\text{NDVI}}_{\text{s}}}{{\text{NDVI}}_{\text{v}}-{\text{NDVI}}_{\text{s}}}$$ (2)

where NDVI_s_ represents NDVI values of bare soil, while NDVI_v_ represents NDVI of fully vegetated areas. This normalization step has the side effect of minimizing errors from atmospheric correction [27, 46]. NDVI_v_ and NDVI_s_ were estimated individually for each date by a histogram analysis for the temporal continuous land cover classes broadleaf forest and bare ground, respectively. Using FVC, the LAI is then calculated with the following function after [47]:

$$\text{LAI}=\frac{-\text{ln}(1-\text{FVC})}{\text{k}(µ)}$$ (3)

where k(μ) is the light extinction coefficient for a solar zenith angle and is a measure of the attenuation of radiation in the canopy. The solar zenith angle (μ) depends on the terrain geometry, solar declination, solar elevation angle, latitudinal location, and the day of the year [42]. Following [48] k(μ) was set to 0.54. Due to bare soil heterogeneity, few pixels with NDVI less than NDVI_s_ result in negative LAI, which were set to 0.

With this generalized approach [49], a dataset is generated that can record the temporal development of spatial heterogeneity for crops in the test area. For more information about this approach, see the study by [46] in the same region.

#### Filtering of pixels for the analysis

The remote sensing scenes were clipped to the extent of the test area. Selection of areas assigned arable land use types resulted in roughly 21.1 million pixels. To avoid border effects, pixels of potentially mixed land use were excluded from the dataset. For this purpose, only pixels surrounded by pixels of the same land use type were selected based on the 15 m resolution land use map. Therefore, the dataset consists of continuous areas with uniform land use separated by borders with no data. The remaining roughly 10.4 million pixels that were assigned an arable land use type are considered the “total arable area”. Since the RapidEye scenes did not always cover the complete test area, the overall number of pixels diverges for some scenes.

#### Remote sensing 5 m resolution dataset (*rs5m*)

A preceding analysis of the remote sensing LAI dataset together with the land use data revealed contradictory combinations of LAI and land use. For example, pixels assigned the land use type maize were found to have a substantial LAI on 2011/02/22 which is even prior to usual sowing dates of maize. Effects like this can be caused by weeds, catch crops or intercrops. For the analysis of separate crops, pixels with LAI values that are impossible for the given land use were excluded. However, they were included when analyzing the overall arable area.

In this study, this dataset is called “*rs5m*” (remote sensing 5 meter resolution, DOI: 10.5880/TR32DB.21).

#### Remote sensing field means dataset (*rsfm*)

For the analysis of mean values for agricultural fields, a spatial aggregation was applied to the *rs5m* LAI maps. The distinct areas of uniform land use that resulted from the exclusion of potential mixed land use pixels (see section on pixel filtering) were defined as individual fields. Therefore, adjacent fields of the same land use cannot be distinguished. This procedure resulted in 24697 spatial entities with field sizes from 25 m² (1 pixel) to 51.9 ha (20745 pixels). In order to use only fields containing enough pixels for statistical analysis, fields smaller than or equal to the median size of 2250 m² were excluded from the analysis. This criterion led to the exclusion of 12380 spatial entities but merely 2.8% of the total arable area. The resulting field dataset consists of 12317 individual fields with a minimum size of 2300 m² (92 pixels). The average field size is 2.06 ha (823 pixels), which is only slightly smaller than the pixel-size in the simulation (2.25 ha). Due to the varying spatial coverage of the remote sensing scenes, data are missing for 45 fields on 2011/02/22 and for 6 fields on 2011/03/03. Analog to the procedure with *rs5m* (previous section) the dataset was filtered to exclude fields whose LAI data contradicts the assigned land use type.

In this study, this dataset is called “*rsfm*” (remote sensing field mean, DOI: 10.5880/TR32DB.22).

### Simulation (*sim*)

#### The DANUBIA simulation system

The DANUBIA simulation system couples models of different complexity and temporal resolution in a component- and raster-based framework. From the 17 (natural and socio-economic) components of the overall system [50, 51], only the components representing plant growth [52], soil nitrogen transformation [53], hydrology, and energy balance (based on [54] and [55]) were used for the current study. These models simulate fluxes of water, nitrogen and carbon in the soil-vegetation-atmosphere system using physically based process descriptions. The models dynamically interact at runtime, which enables DANUBIA to include numerous feedback effects. Further information on the open source DANUBIA simulation system is available at www.glowa-danube.de.

#### Crop growth in DANUBIA

The crop growth model [52] simulates fluxes of water, carbon, and nitrogen within crops and an energy balance at the leaf level. Main processes included are photosynthesis, respiration, soil layer-specific water and nitrogen uptake, dynamic allocation of carbon and nitrogen to four plant organs (root, stem, leaf, harvest organ), and phenological development and senescence. The model is based on GECROS [56] and CERES [57] with extensions from [58, 59] for modeling phenological development.

The model differentiates green LAI from total LAI. Green LAI is calculated as the minimum of a carbon-limited and a nitrogen-limited LAI [60, 61]. Senescence reduces green LAI if leaf nitrogen content falls below a minimum value.

For a validation of LAI, aboveground biomass and soil moisture in DANUBIA refer to [52] and [32].

#### Input data and model runs

In this study, DANUBIA was run with 150 m spatial resolution for areas assigned land use maize, winter wheat, or sugar beet in the test area for the cropping season 2010/2011. Crop-specific model parameters were mainly set as presented by [52]. Crop management dates and amounts of fertilizer were set uniform throughout the catchment.

Some model parameters were adjusted in a calibration step in order to make the general timing of the temporal development of LAI consistent with field measurements and remote sensing results. Deviations from the original parameterization [52] are given in Table 2. Spatially uniform agricultural management activities were presumed (Table 2).

**Table 2:** DANUBIA parameterization. Parameter values deviating from the original setting [52] and cultivation information.

crop	Parameterization		cultivation		
	parameter	Value	activity	date	amount
maize	weight per seed	0.38 g	sowing	2011/04/25	
	specific leaf area	0.0295 m² g^-1^	fertilization	2011/04/26	170 kgN ha^-1^
	senescence	25 %	harvest	2011/09/26	
sugar beet	seed N concentration	0.002 g g^-1^	sowing	2011/03/23	
	specific leaf area	0.019 m² g^-1^	fertilization	2011/03/24	100 kgN ha^-1^
			fertilization	2011/04/14	60 kgN ha^-1^
	initial biomass	28.5 g m^-2^	harvest	2011/11/17	
	senescence	10 %			
winter wheat	sowing depth	1.5 cm	sowing	2010/10/10	
	seed N concentration	0.2 g g^-1^	fertilization	2011/08/15	75 kgN ha^-1^
			fertilization	2011/05/04	40 kgN ha^-1^
			fertilization	2011/05/30	80 kgN ha^-1^
			harvest	2011/08/15	

Spatially explicit data on land use (see above), soil (1:50000) [62], and terrain (digital elevation model) [63] were taken from the project’s database (www.tr32db.de). Data were upscaled to the 150 m grid if necessary. Meteorological drivers were interpolated to the 150 m grid applying the method described by [54] using measurements from 19 stations of the German National Weather Service within or in direct proximity (<20 km) to the Rur catchment. Measured precipitation was corrected according to Richter [64]. The model run started on 2010/08/01. Initial conditions of the soil were set based on supplementary data from the soil map.

In this study, the simulation dataset is called “*sim*” (simulation, DOI: 10.5880/TR32DB.23).

### Analysis of spatial heterogeneity

Following the concept of [65] spatial heterogeneity is characterized by spatial variability and by spatial structure. In the current study, standard deviations and relative frequency distributions are used to analyze the temporal development of spatial variability. To analyze spatial structure, semivariograms were calculated. All analyses were performed separately for maize, sugar beet, winter wheat and for the overall arable area.

Removal of pixels of potential mixed land use (section on pixel filtering) changed the relative abundance of the crop types. This would lead to a bias in the analysis of spatial variability of the overall arable area. Thus, mean values and relative frequency distributions were calculated as weighted means of the results for the separate crops in order to avoid this potential bias. Overall standard deviations were derived by calculating the square root of the pooled variance.

### Accordance of relative frequency distributions

To quantitatively compare relative frequency distributions, a measure of similarity or accordance is required. In this study, the accordance *a* was derived by calculating the overlap of the distributions (histograms):

$$a=\sum_{c=1}^{n_{c}}\text{min}\left(D_{a,c},D_{b,c}\right)$$ (4)

where D_a_ and D_b_ are two relative frequency histograms with n_c_ classes of identical class width; *c* is the class index. The value of *a* represents the fraction of values that fall in the same class. Accordance depends on the class width of the frequency distributions. Below a certain class width, values of *a* stay constant. Therefore, in the current study, accordance was calculated using a class width of 0.1 LAI.

### Geostatistical analysis of spatial structure

Geostatistical analysis for *rs5m*, *rsfm* and *sim* was conducted using the R-package gstat [66]. Experimental semivariograms were generated with a class width of 200 m until a maximum lag of 25 km (about half the maximum distance in the test area). For 5 m resolution remote sensing data (*rs5m*), the number of data points is too large for the analysis. Thus, in order to achieve reasonable computing times, 0.5% of the data points were randomly selected. Repeated execution of this step (10 times) resulted in a maximum per-lag-class deviation of 1.7% from the mean. Thus, a fraction of 0.5% is a sufficient number of datapoints for the analysis.

To analyze the spatial structure of the overall arable land, prior to the calculation of semivariograms, the relative abundances for the different crops were adjusted to match those given by the original land use classification. This was achieved by randomly removing pixels (*rs5m*) or fields (*rsfm*) of land use types with a too high abundance.

Most semivariograms generated from *rs5m*, *rsfm*, and *sim* have shape that cannot be approximated by any of the usual theoretical semivariogram models. Therefore, a nested semivariogram model consisting of the sum of two exponential functions was fitted to the data. If the fitting process resulted in an error or a singularity, various sets of start values for sills and ranges were tested. In addition, various fitting methods and exclusion of the nugget model were analyzed. If fitting was still not possible or if the nested model did not fit the transition to the plateau, a simple exponential model was used or the semivariogram was cut at a maximum distance of 6 km. Geostatistical assessment was not applicable for *field* data since the number of data per field is too small.

## Results and Discussion

The current study analyses (green) LAI spatial heterogeneity and its temporal development. Prior to the analysis of LAI spatial heterogeneity, the consistency of the analyzed datasets is examined.

### Consistency

To ensure that results of the simulation (dataset *sim*) and from remote sensing (dataset *rs5m* in 5 m resolution and dataset *rsfm* with mean values for agricultural fields) were plausible, they were checked against the field measurements (dataset *field*).

Since the test area has very favorable conditions for arable farming, maximum LAI is very high (Table 3). Similar values were reported in the literature (maize: [67,68]; winter wheat: [69,70]; sugar beet: [71]). The maximum LAI in *sim*, *rs5m* and *rsfm* was generally lower than in *field* (Table 3). The only exception occured for maize in *rs5m* where the maximum LAI of 6.3 exceeded the maximum of 5.8 in *field*. However, only less than 1% of the maize pixels in *rs5m* exceeds an LAI of 4. In addition, a maximum LAI of 6.3 is still within the range measured for maize [68].

**Table 3:** Maximum LAI of the three main crops from field measurements (*field*), simulation (*sim*) and remote sensing (*rs5m* 5 m resolution, *rsfm* means for agricultural fields).

	*field*	*sim*	*rs5m*	*rsfm*
maize	5.8	5.3	6.3	4.5
sugar beet	9.5	6.6	7.0	5.6
winter wheat	8.9	7.3	5.4	4.0

Since there is no cloud-free remote sensing scene available between 2011/04/07 and 2011/06/27, the remote sensing data do not cover the period of maximum LAI for winter wheat. Therefore, in both remote sensing datasets, the maximum LAI for winter wheat shows the largest differences to *field*. Remote sensing LAI for maize and sugar beet achieved its maximum on 2011/06/27, which is in the beginning of the period of maximum LAI for these crops.

**Fig 2:**
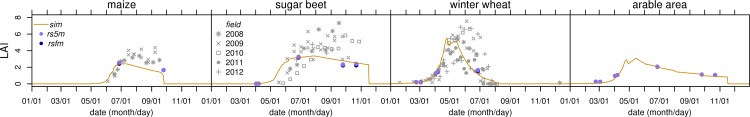
Mean LAI temporal course. Temporal course of the mean LAI of single agricultural fields (*field*, gray symbols) and of the test area from *sim*ulation and remote sensing (*rs5m*: 5 m resolution, *rsfm*: mean LAI of agricultural fields) for three separate crops and for the overall arable area. There is no *field* data for the arable area since the measured fields were too few and thus not representative for the test area.

Maximum LAI in *rs5m* is 7. Previous studies suggest that derivation of LAI from NDVI is not possible for LAI larger than 6 due to saturation of the dependency [26–28]. In *field*, LAI greater than 6 occurred for winter wheat between the end of April and the end of June and for sugar beet from June on (Fig 2). Therefore, the remotely sensed LAI might be underestimated for winter wheat on 2011/06/27 and for sugar beet on 2011/06/27, 2011/09/25 and 2011/10/22. However, LAI greater than 6 were derived in *rs5m* only on 2011/06/27. On that date, the 95th percentile is at an LAI of 4.1 for sugar beet and at 2.4 for winter wheat. Thus, only few pixels are potentially affected by the saturation effect.

The analysis of the temporal course of mean LAI for *field* (means over all datapoints) and for *sim*, *rs5m* and *rsfm* (mean over the test area) reveals almost identical values for *rsfm* and *rs5m*, a good agreement of the remote sensing datasets with *sim*, and some diverging properties of *field* (Fig 2).

For **maize,** there are only two dates in the remote sensing datasets. On 2011/06/27, the mean LAI matches with *sim*. On 2011/09/25, it is slightly higher because of an earlier beginning of senescence or a too low peak LAI in the simulation. The temporal course of *field* shows similar characteristics but the increase of LAI starts later than in *sim* and reaches higher values.

For **sugar beet**, similar to maize, *sim* shows an earlier increase of LAI and a lower maximum LAI than *field*. *sim* and the remote sensing datasets show similar mean values. The only clear difference is on 2011/09/25. While *rs5m* and *rsfm* exhibit a slight increase of mean LAI from 2011/09/25 to 2011/10/22 *sim* shows a slight decrease. This is also the case for most fields in *field*. The offset might be caused by a general offset in the remote sensing data on one of the days. This might have been introduced to the data by using an uncalibrated approach to derive LAI from reflectivity. However, since these differences are much smaller for the overall arable area, there is no evidence for a general error.

As for the other separate crops, mean LAI of **winter wheat** in *sim* shows good agreement with the remote sensing data but has lower values during senescence on 2011/06/27. Especially the concurrent increase of LAI from 2011/04/02 to 2011/04/07 affirms the assumption that green LAI is detectable from RapidEye NDVI by the approach used in the current study. In the early season, the increase of LAI is similar to *field*. After mid-April, increase in *sim* is stronger and saturates earlier and at a lower maximum LAI than in *field* data of most years. However, *field* measurements from 2011 (gray dots in Fig 2) show a temporal course similar to *sim*.

In general, two effects may explain the higher mean LAI in *field* for the separate crops. Firstly, the data for *field* is from a few fields with very favorable growing conditions, while the other datasets include regions with less favorable soil conditions and therefore show lower values. Secondly, since the remote sensing data are derived from 5 m resolution scenes, there is already an averaging effect as compared to *field*, which represents one square meter. In addition, remotely sensed LAI can be underestimated for a rough canopy top due to shading effects. This especially applies to crops like maize with a large distance between individual plants.

For the overall **arable area**, results show good agreement of *sim* and the remote sensing datasets (RMSE 0.16). Additional crops, catch crops and weeds are not included in *sim*. This can explain the higher mean LAI of the remote sensing datasets in the early season. Sudden changes of the mean LAI of *sim* in the end of July and September and in mid-November (Fig 2) are caused by the simultaneous harvest of all pixels of a given crop in the simulation. In reality, transitions will be smoother because of limitations of farm operations (e.g. availability of harvesters) and differences in the date of maturity due to different sowing dates.

From the analysis above, it can be concluded that the datasets used in the current study are sufficiently consistent to be combined in order to analyze the temporal course of LAI spatial heterogeneity.

### Spatial variability

At first, the analysis of spatial heterogeneity was focused on spatial variability. A simple measure for spatial variability is the **standard deviation (SD)**, since it describes the scattering of the data around a mean with a single number. The analysis was done separately for intra-field variability and overall variability of the test area.

**Fig 3:**
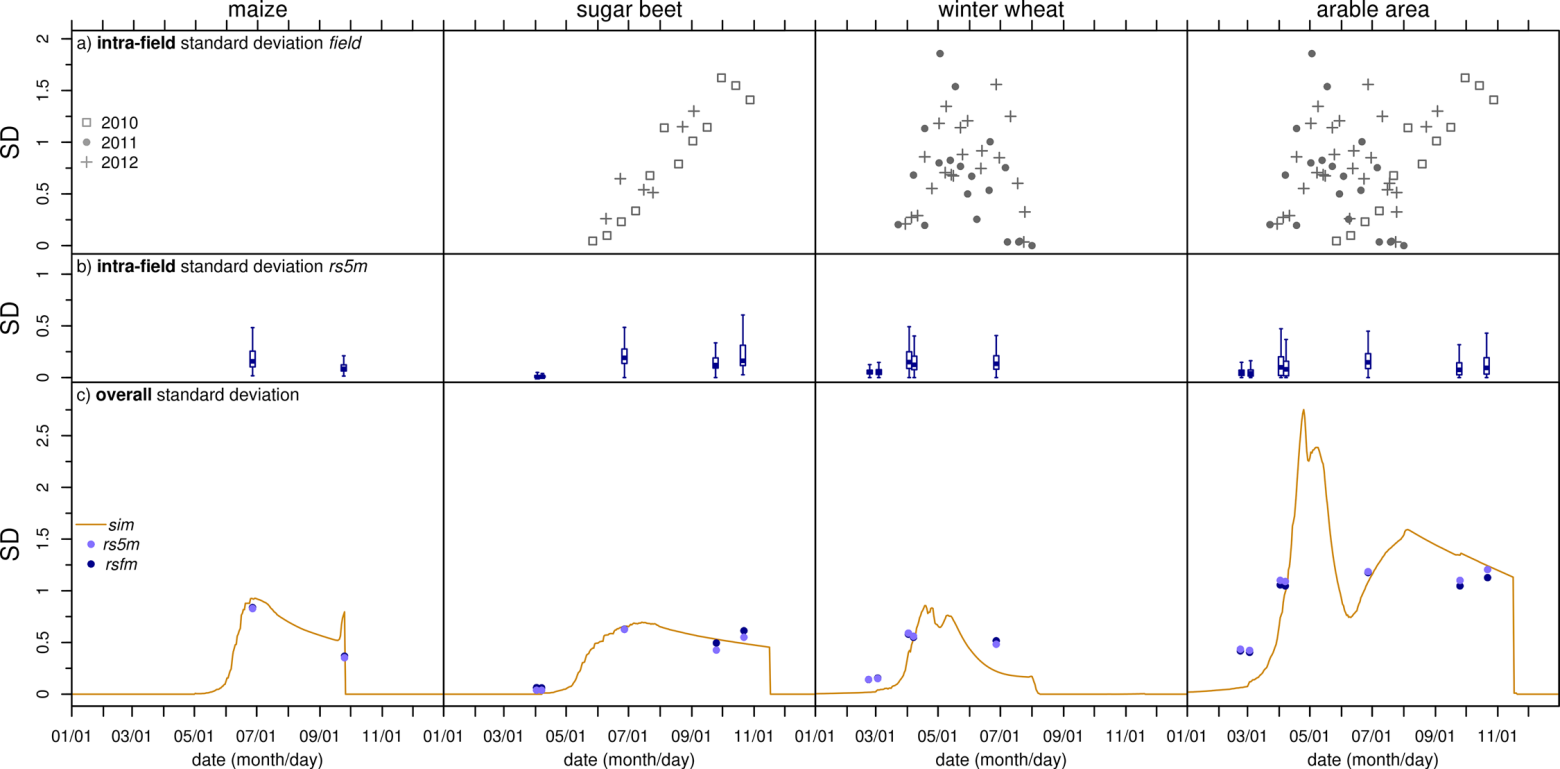
Spatial variability: standard deviations temporal course. Temporal development of LAI spatial variability expressed as standard deviation (SD) for three separate crops and the overall arable area. (a) Intra-field SD from *field* measurements only includes fields with at least six measurement points, (b) intra-field SD from 5 m resolution spaceborne remote sensing (*rs5m*) as boxplots with whiskers ranging to 1.5 times the interquartile range, (c) standard deviations over the test area for the simulation (*sim*), *rs5m*, and remote sensing field means (*rsfm*) derived from *rs5m*. Each for three separate crops and for the overall arable area of the test region. Y-axes have the same scale and are clipped to save space.

**Intra-field SDs** (Fig 3A and 3B) in the *field* data were calculated for fields with at least six measurements per observation date. Therefore, there is no data for maize. Intra-field SDs in *rsfm* were calculated for each field (for a definition of a “field” see section on remote sensing dataset). The latter is presented as boxplots with whiskers ranging to 1.5 times the interquartile range (Fig 3B). Thus, erroneous high intra-field SDs originating from areas not cultivated uniformly but interpreted as fields are excluded.

The temporal development of *field* intra-field SD of the different years and sites (Fig 3A) exhibits shifts of the patterns in time. These shifts show differences in phenological development, mainly caused by varying weather, management and crop cultivar.

Intra-field SD in *rs5m* mostly shows lower values than that from *field*. This can be attributed to the much finer spatial resolution of the point measurements in the field compared to the 25-m² pixels of *rs5m*. Even at this resolution of 5 m, the averaging effect reduces variability. Intra-field SDs from both datasets show a temporal development qualitatively similar to that of the mean LAI with higher SD at higher LAI.

**Overall SDs** of the different datasets are generally in good agreement (Fig 3C). Differences occur in the early season where *sim*, in contrast to the remote sensing datasets, shows uniformly low LAI. For sugar beet, SD from *sim* is higher on 2011/09/25 (more pixels with high LAI in *sim*) but lower on 2011/10/22 (converging LAI in *sim* due to browning of leaves). The deviation of *sim*-SD from remote sensing SD for the overall arable area on 2011/09/25 is caused by a narrower distribution of *sim*-LAI and much smaller fractions of pixels with LAI between 0.1 and 2.3 (see section on frequency distributions below).

The smallest differences of overall SDs occur between *rs5m* and *rsfm*. Thus, some points in Fig 3C occupy almost the same location and are therefore hard to distinguish. Since the averaging effect of generating *rsfm* from *rs5m* is small, it can be concluded that in the test area, inter-field variability dominates over intra-field variability. This is confirmed by the fact that overall SDs are higher than the majority of intra-field SDs in the remote sensing data (Fig 3).

SD of the overall arable area is higher than that of the separate crops. Since the mean LAI of the overall arable area and the separate crops is of the same magnitude (Fig 2), the overall arable area shows higher relative variability than the separate crops. The variability of LAI for the separate crops is mainly caused by local and regional differences in the plant growth driven by soil and weather variability. The variability of the overall arable area is higher due to the differing seasonal LAI development of different crops.

For the separate crops, the temporal course of the overall SD (Fig 3C) resembles that of the mean LAI (Fig 2). In contrast, for the overall arable area, the temporal courses of mean LAI and SD differ. While mean LAI on 2011/06/27 is about twice the mean LAI on the two precedent and subsequent remote sensing dates (Fig 2), SD is similar on all of these dates (Fig 3C). As can be seen from *sim* data, larger differences exist in the intervals between the remote sensing dates. Until June, SD is dominated by the contrast between increasing winter wheat LAI and LAI close to zero of maize and sugar beet. While the mean LAI declines continuously thereafter, the temporal course of the SDs shows a local minimum (in early June) and maximum (in early August) that are not present for the mean LAI. The decline of SD occurs because winter wheat LAI declines at the same time as sugar beet (and later maize) LAI increases. This convergence causes LAI of the different crops to be more similar in early June, which results in a minimum of the SD. After the local minimum, LAI of the crops diverges again causing SD to increase until winter wheat is harvested in early August. Since then, winter wheat LAI in *sim* is constantly at zero and the decline of the overall SD is determined by the combined decreasing SDs of sugar beet and maize.

After harvest and until mid-march, *sim* SD is close to zero because in the simulation LAI is less than 0.5 for all of the crops. *rs5m* and *rsfm* show higher SDs for the overall arable area in February and March denoting higher variability. This additional variability is generated by fields with catch crops and weeds, which are not included in the model. This also bears consequences for SD during the growing season where the fraction of pixels with an LAI of zero in *sim* has an increasing effect on the overall SD of *sim*. The LAI of catch crops or weeds is higher and therefore has a less increasing effect on the overall SDs of *rs5m* and *rsfm*. If catch crops and weeds were included in the simulation, overall SD of *sim* would be lower than that of the remote sensing datasets.

**Fig 4:**
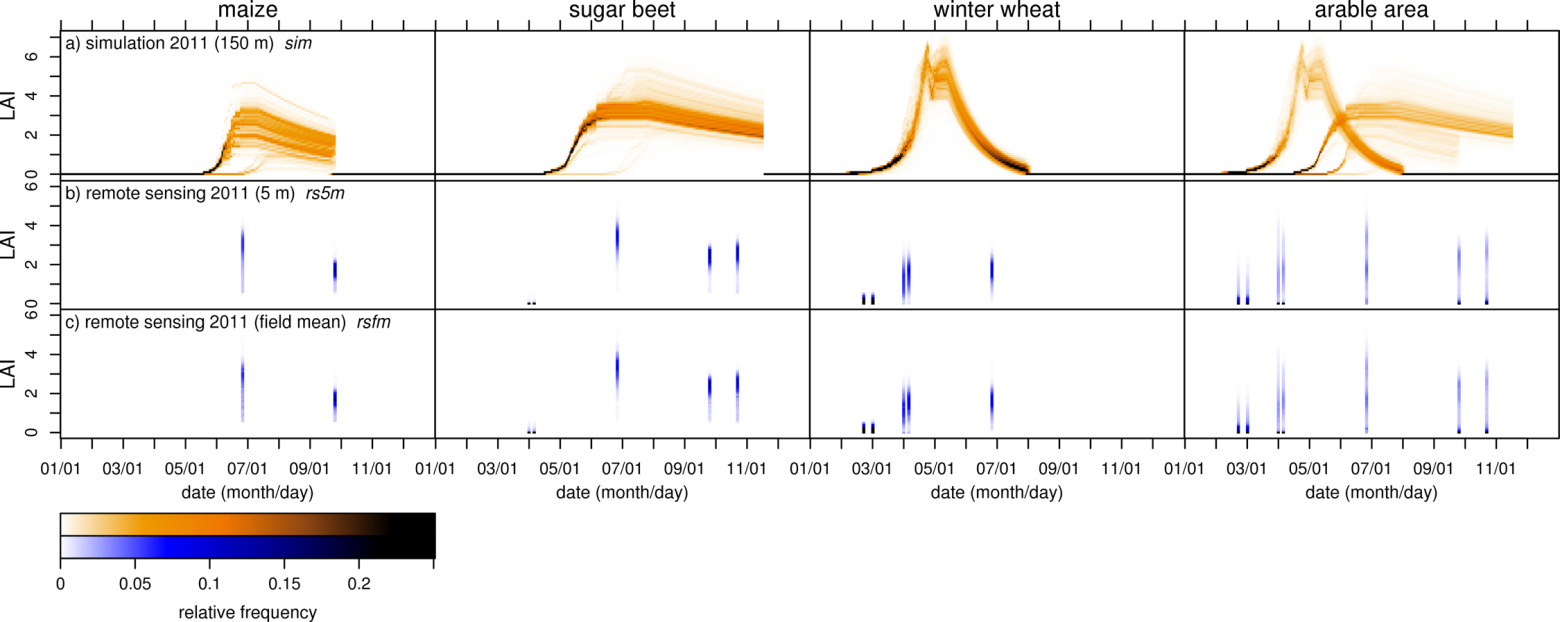
Spatial variability: relative frequency distributions temporal course. Temporal development of LAI spatial variability expressed as (vertical) color-coded relative frequency distributions (RFD). (a) RFD of simulated LAI (*sim*), (b) RFD of LAI derived from 5 m resolution spaceborne remote sensing (*rs5m*), (c) RFD of LAI field means (*rsfm*) derived from *rs5m*. Each for three separate crops and for the overall arable area of the test region. Width of LAI classes in the RFDs is 0.1.

Knowledge about the LAI development for the separate crops is needed to explain the SD for the overall area. While for the current study, the detailed land use classification provided detailed information about the crops, for studies on a coarse scale only frequency distributions of the LAI for the overall area might be available. Fig 4 shows the **relative frequency distribution** (RFD) of the LAI for the separate crops and for the overall arable land.

The temporal course of RFDs for *sim* (Fig 4A) shows a characteristic pattern for each of the three crops. **Maize** LAI starts to increase in mid-May. In early June, LAI begins to diversify causing a broader RFD. After the time of maximum LAI when senescence begins, spatial differences in LAI decrease again and the RFDs become narrower. Until end of June, a fraction of grid cells with very low LAI exists due to very low soil moisture. This enhanced variability causes a steeper increase of SD until early June. For **sugar bee**t, LAI starts to increase in mid-April. After a period of narrow distributions, LAI begins to diversify in late May. Maximum LAI occurs between late June and mid-July followed by a period of slight senescence. Similar to maize, RFDs become narrower during senescence. In addition, there also is a fraction of very low LAI, which causes enhanced variability until early June. **Winter wheat** LAI starts to increase early in the growing season. Until the first peak of LAI, RFDs are narrow. During the subsequent stress-induced decrease and re-increase of LAI, RFDs widen. After mid-May, LAI declines rapidly. RFDs become narrower resulting in about 20% of all LAI values in the same histogram class (dark color in Fig 4A).

Against the backdrop of the crop-specific temporal patterns, the patterns of the RFDs for the overall **arable area** of *sim* (Fig 4A, column on the right) can be understood as a superposition of the three separate crops’ RFDs. In the beginning of the year, variability is generally low (LAI<0.3 until beginning of March). It increases when winter wheat starts to grow in the beginning of March. From this time on, there is more than one peak in the RFD. The type of graph used here clearly shows the multimodality of the RFD. When using SD or quantiles (as in boxplots) this information would be lost. A considerable fraction of pixels with low LAI that exists until maize starts to grow in mid-May causes a higher variability. Starting in mid-April, the frequency distributions show multiple local maxima (parallel dark “areas” in Fig 4A), thus indicating increasing variability. While winter wheat LAI is at its optimum in late April and May, LAI of sugar beet and (later) maize start to increase. In the beginning of June, LAI of both main crops (winter wheat and sugar beet) is in the same range. This is due to the coincidence of senescence of winter wheat LAI and the increase of sugar beet LAI. This causes a lower overall variability, which is also visible in the SD (Fig 3C). Subsequently, variability increases again in late June and July. The contribution of maize to the overall pattern is small because of its smaller acreage. Similar overall patterns were found for the years 2008 and 2009 (not shown). The same analysis using point data of *field* (not shown) confirms these patterns. The patterns found here depend upon the phenological development of the different crops. A study done in Mediterranean France did not show an overlap of the temporal course of LAI for maize and wheat [72]. Therefore, it has to be emphasized that the temporal patterns described in this study are valid only for regions with the same combination of main crops and the same management practice and phenological development of the crops, respectively.

Due to the temporal gaps and due to the dates of the remote sensing scenes, many significant characteristics of the temporal patterns of variability found in *field* and *sim* cannot be seen clearly in results using the remote sensing data (Fig 4B and 4C). This applies especially to the period of exponential growth for maize and sugar beet and to the LAI peak and onset of senescence for winter wheat. Nevertheless, knowing the characteristic temporal patterns, RFDs found for *rs5m* (Fig 4B) and *rsfm* (Fig 4C) can be identified as temporal snapshots thereof. In particular, the increase of LAI from 2011/04/02 to 2011/04/07 as well as the noticeable fractions of LAI close to zero for the overall arable area and the triple peak on 2011/06/27 show that simulation and remote sensing capture the same properties of the temporal development of LAI spatial variability. As described earlier in the context of dataset consistency, a mismatch of the datasets exists for sugar beet in the late season where LAI increases in *rs5m* and *rsfm* whereas *sim* and *field* show a slight decrease (discussed in section on consistency).

**Fig 5:**
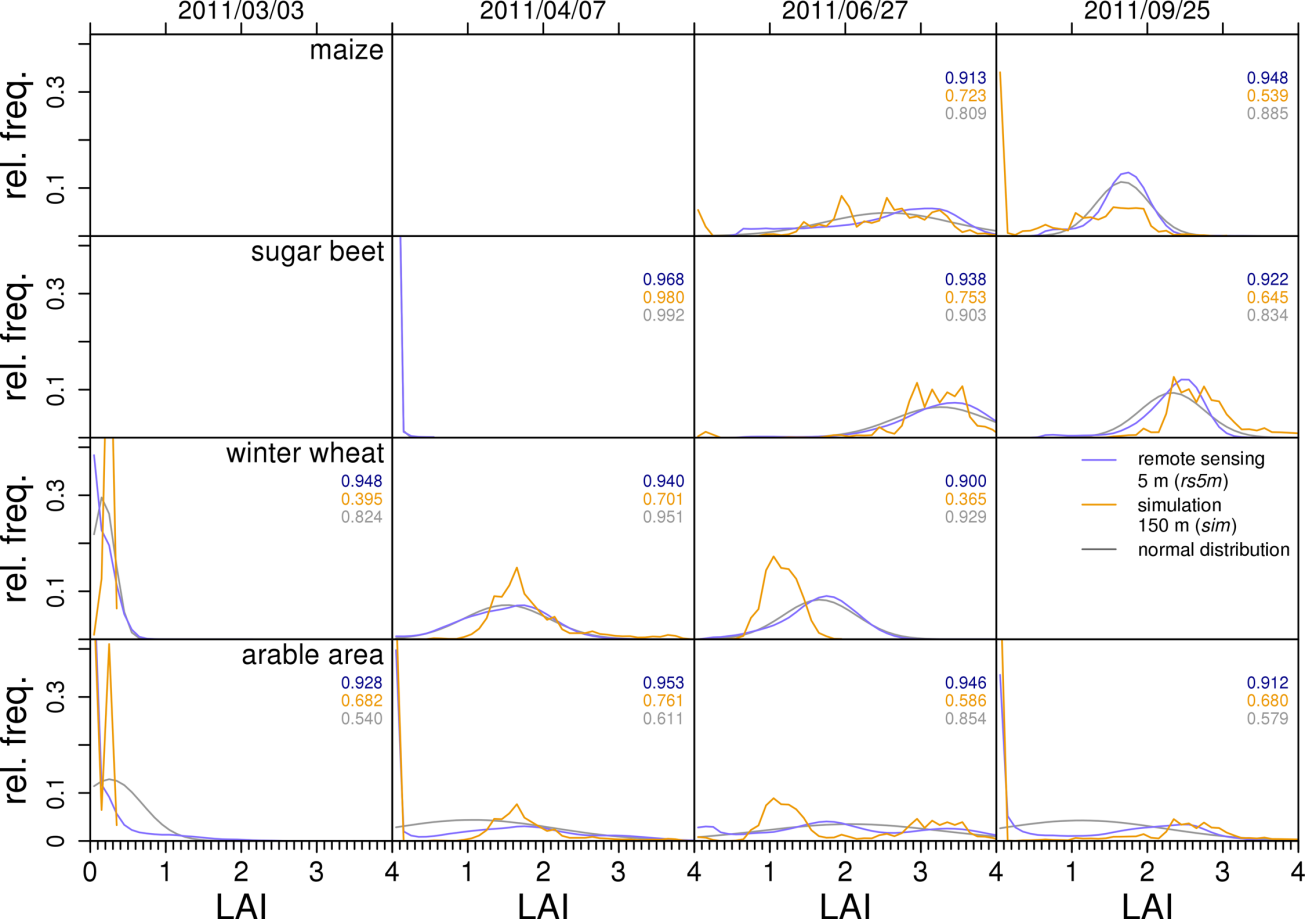
Spatial variability: comparison of relative frequency distributions for selected dates. Histograms of the relative frequency distributions (RFD) of green LAI from simulation (*sim*), 5 m resolution remote sensing (*rs5m*), and from a normal distribution generated from mean and standard deviation of *rs5m*. Each for three separate crops and for the overall arable area of the test region for four selected dates of remote sensing scenes (overpass dates of RapidEye). Width of LAI classes in the RFDs is 0.1. Both axes are clipped to improve readability. Outside of the growing season, there is no data for separate crops. The numbers denote the accordance of frequency distributions between *rs5m* and *rsfm* (top, blue, data not presented in the figure), *rs5m* and *sim* (middle, yellow) and *rs5m* and a normal distribution (bottom, gray).

For a more detailed **comparison of datasets**, RFDs are shown as histograms in Fig 5. Since frequency distributions for nearby remote sensing dates are similar, data from 2011/02/22, 2011/04/02 and 2011/10/22 are not shown. Histograms are presented using lines instead of the usual bars to enable an easier comparison of datasets. In order to compare histograms, the overlapping fraction of the frequency distributions (4) is used as a measure of accordance.

Like their SDs, RFDs of ***rs5m* and *rsfm*** are in very good agreement. The accordance is generally above 0.9 (uppermost, blue numbers in Fig 5). Therefore, data of *rsfm* is not presented in Fig 5. RFDs of *rsfm* have their maximum frequency at the same or slightly lower LAI than *rs5m*. The small differences between the datasets confirm the finding that the overall variability is dominated by inter-field variability.

RFDs of ***sim* and *rs5m*** differ in timing and extreme values as described in the section on consistency. Additional differences can be seen when comparing the RFDs. Maximum frequency of *sim* is typically at an LAI lower than that of *rs5m* and *rsfm*. Peaks of RFDs of *sim* are typically narrower and show higher frequencies. In agreement with theory, this demonstrates a lower variability in the lower resolution *sim* data compared to the remote sensing datasets. As can be seen from the example of maize, a second peak close to zero in the RFD of *sim* may superimpose this by generating lower relative frequencies at higher LAI values. Here, differences in the timing of crop growth (early senescence in the simulation) cause differences in variability.

The RFDs also depict the multiple local maxima for s*im* in more detail and show the difference to the RFDs of *rs5m*, which are always continuous. The multiple local maxima are an effect of the discretization of soils and crops in the model. In reality, soil properties are a continuum and crop properties are diverse between individuals of a species. In the model, soil properties of each pixel are set according to a certain texture class. Therefore, each pixel that belongs to a certain soil texture class is assigned the same soil properties causing discrete spatial units with uniform soil properties. On these soils, the model simulates plants with properties and management settings that are uniform for a specific crop. The resulting discrete soil-crop combinations in turn generate a number of discrete peaks in a crop’s RFD. Only the weather, which is unique on each pixel, introduces a kind of smearing effect that turns RFDs of *sim* from separate spikes to the shape shown in Fig 5.

The discrepancies in the shapes of the RFDs of *sim* and *rs5m* result in rather low accordance values (yellow numbers in Fig 5). Except for sugar beet in the early season, they are between 0.54 and 0.76. The lower value of accordance of *sim* and *rs5m* compared to that of *rsfm* and *rs5m* has multiple reasons: (1) Because of the several local maxima, RFDs of *sim* are less continuous than those of *rs5m* are. (2) LAI ranges in *sim* are narrower and typically show higher frequencies than those of *rs5m*. (3) *sim* and *rs5m* differ in timing (e.g. winter wheat on 2011/06/27).

The description of a specific RFD requires much data. In the following, it is tested whether a much simpler description with mean and standard deviation expresses the RFD sufficiently well. This should be the case when there is only a single peak in an RFD and the RFD is rather symmetric as it is the case for the separate crops. To test this, based on mean and SD of *rs5m*, **normal distribution**s were generated (grey curves in Fig 5). The accordance of the original RFD for *rs5m* was calculated (grey numbers in Fig 5). For the separate crops, these accordance values are always higher than the accordance values for *rs5m* and *sim*. Thus, taking *rs5m* as a reference, the normal distribution is a better description of LAI variability for the separate crops than the simulation results. In case of the overall arable area, RFDs of *sim* show higher accordance values with *rs5m* than normal distributions do. Only when there is no peak at zero (2011/06/27), does the normal distribution have a higher accordance value. On that date, the major cause for the lower accordance is the too low LAI of winter wheat. A better timing of senescence in the simulation should increase accordance. However, for the overall arable area, despite of the differing spatial resolutions, simulation results are mostly better descriptions of variability than normal distributions.

### Temporal development of LAI spatial structure

Spatial structure is the second aspect of spatial heterogeneity. In this study, it is analyzed utilizing semivariograms. The *field* dataset was excluded from this analysis due to the small number of points per field (max. 12).

**Fig 6:**
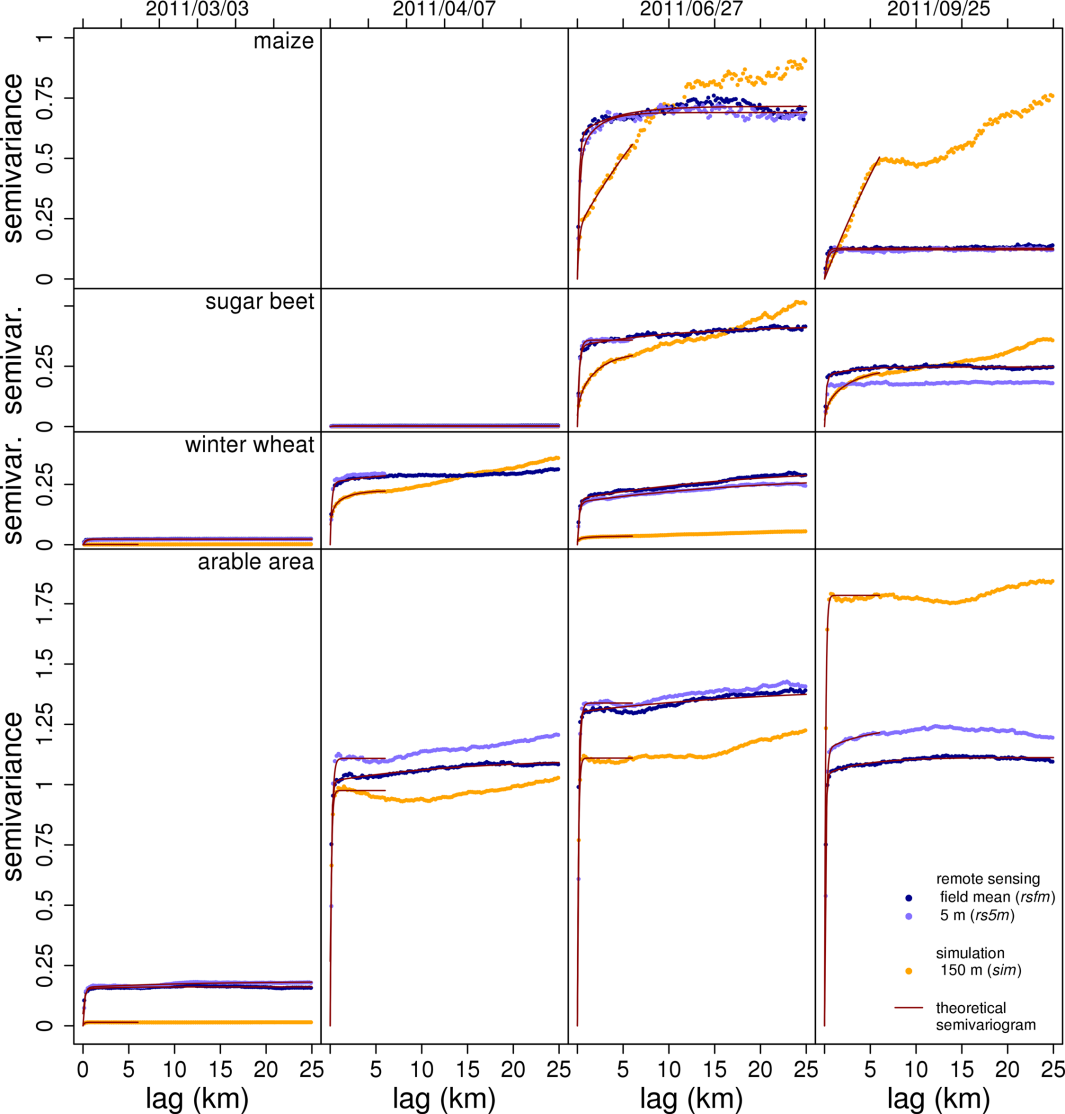
Spatial structure: semivariograms for selected dates. Spatial structure of green LAI expressed as semivariograms from simulation (*sim*), 5 m resolution spaceborne remote sensing (*rs5m*), and from LAI field means (*rsfm*) derived from *rs5m*. Each for three separate crops and for the overall arable area of the test region for four selected dates of remote sensing scenes (overpass dates of RapidEye). Experimental semivariograms are shown as dots and theoretical semivariograms as lines. There is no data for dates outside the growing season of the respective crop. Y-axes have the same scale and are clipped to save space.

The semivariograms derived from the remote sensing images show a steep initial increase of the semivariance and subsequently either a slow gradual increase or a more or less constant semivariance. The semivariance calculated from the simulation for the **separate crops** shows a more gradual initial increase and in most cases a larger variability and a steeper increase at larger lags as compared to the remote sensing data results (Fig 6). The smaller initial slope derived for the simulation results is due to higher autocorrelation on short lags which in turn is due to the very similar LAI of pixels in close vicinity. As discussed in the section on spatial variability, this is probably caused by the reduced variability of soil, crop properties, and weather data in the model.

Semivariograms calculated from the remote sensing data (*rs5m* and *rsfm*) for the **overall arable area** show a very clear edge of the initial increase to the plateau. Most semivariograms show a slight increase at longer lags. Thus, even for the virtually homogeneous agricultural landscape investigated here, on large scales the variability increases due to large scale differences of climate, soil or topography. As opposed to the individual crops, the course of the semivariograms for *sim* for the overall arable area is very similar to that derived from remote sensing data. Thus, the effect of the reduced heterogeneity of soil and weather in the simulation, which led to the smaller initial slope for the separate crops (intra-crop variability), is masked by the variability introduced by fields with different crops (inter-crop variability).

**Fig 7:**
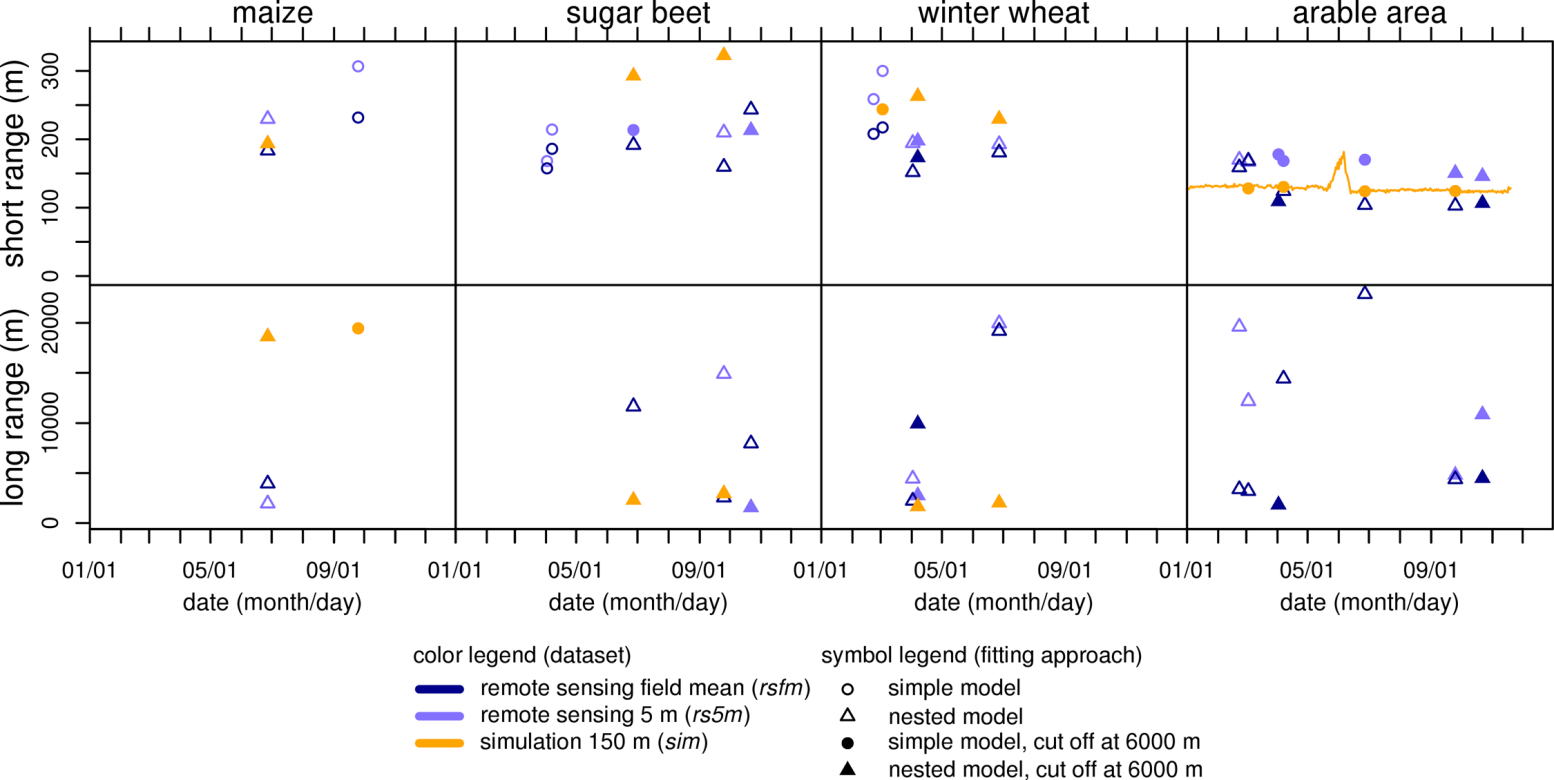
Spatial structure: temporal course of semivariogram ranges. Ranges of fitted exponential semivariogram models from simulation (*sim*), 5 m resolution remote sensing (*rs5m*), and from LAI field means (*rsfm*) derived from *rs5m*. Each for three separate crops and for the overall arable area of the test region for four selected dates of remote sensing scenes (overpass dates of RapidEye). Symbols denote whether fit was accomplished using a simple model, a nested model, or a simple model cut off at lags of 6000 m.

To address the spatial structure quantitatively, a theoretical semivariogram model was fitted. The semivariogram’s range is the lag where the semivariance of LAI does not increase for longer lags and thus the initial increase turns into a plateau. It is a measure for spatial structure since it tells the length scales of the spatial entities in the area [65]. Due to the very steep initial slope and the abrupt transition to the plateau in most semivariograms of the current study, it is crucial to get a good fit in the transition zone to determine the range properly. Irregular shapes of the semivariograms often required diverging from the simple exponential model (filled circles in Fig 7) by (i) fitting a nested model consisting of the sum of two exponential models (open circles in Fig 7); (ii) using differing settings for the fitting algorithm (start values, weighting factors, not marked in Fig 7); or (iii) cutting off the semivariogram at distances of 6000 m (crossed circles in Fig 7). The effect of the different fitting approaches on the derived semivariogram ranges was tested by fitting a simple cut off model and a nested model to the same datasets. This test resulted in a mean change of the short ranges of 8 m. Their upper boundary of the short ranges increases to 376 m. From these small changes, it is concluded that fitting of the nested model does not qualitatively change results for the short ranges. Fitting of nested models (open circles in Fig 7) results in two ranges that describe two independent effective lengths. The analysis of the fitted models reveals two groups of ranges with distances below 325 m (short ranges) and above 1560 m (long ranges), respectively (Fig 7).

For the **separate crops**, the **short ranges** are between 150 and 320 m (Fig 7). These distances correspond to the dimensions of arable fields in the test region. Despite the different shapes and fitting settings, there are systematic differences between the datasets. Except on 2011/10/22 for sugar beet, ranges of *rs5m* are always longer than those of *rsfm*. In addition, *sim* ranges in most cases are longer than those in both remote sensing datasets. Presumably, *rs5m* shows longer short ranges than *rsfm* because of the intra-field heterogeneity, which introduces additional variability at the short lags elongating the distances at which LAI values are dissimilar. The even longer short ranges for *sim* are due to the much lower spatial resolution of the dataset and to the high similarity of LAI on nearby fields caused by the reduced variability of model input (see above).

Short ranges for the **overall arable area** are between 100 and 180 m and are thus shorter than those for the separate crops are. This is due to the origin of spatial variability. For the separate crops, spatial variability originates from intra-crop variability. For the overall arable area, inter-crop differences in LAI development and thus differences between different crops, produce an additional source of variability (note the higher sills in Fig 6). Inter-crop variability is usually larger than intra-crop variability (Fig 5). Since there are different crops on adjacent fields for the overall arable area, differences in LAI occur at shorter distances.

The temporally continuous dataset *sim* has the most irregular shaped semivariograms. Thus, fitting of semivariogram models was not feasible for many dates. As a result, the temporal course of the change of the effective length is only shown for short ranges of the overall arable land in Fig 7. Over most of the time, short ranges are between 120 and 130 m. Only in the period between 2011/05/20 and 2011/06/14, do ranges reach distances of up to 180 m. This is the period where the convergence of the LAI of the separate crops (cf. section on spatial variability) causes reduced inter-crop variability. This higher similarity causes ranges to increase.

The **long ranges** are between 1.5 and 22.9 km. They are attributed to influences of soil and weather on LAI development. Analyzing the spatial structure of soil moisture for the same catchment, similar ranges were found [73]. For the long ranges, there is no temporally continuous information since the irregular shape of the *sim* semivariograms does not always allow being fitted to a nested model. Moreover, a pattern in the temporal course of the long ranges was not determined.

## Summary and Conclusions

Referring to hypothesis H1, results from remote sensing (*rs5m* and *rsfm*) and from the simulation (*sim*) were determined to be within the ranges reported in the literature. The temporal course of spatial variability inferred from the simulation is confirmed by the temporal course of *field* measurements. The datasets are therefore suitable for use in the Comprehensive Data Analysis Approach (CDAA) to analyze the spatial heterogeneity of LAI.

By applying the CDAA, a comprehensive picture of LAI spatial heterogeneity and its temporal development was generated. Complex patterns in LAI frequency distributions from remote sensing were explained based on simulation results and differentiation by crop. Moreover, the temporal development of spatial heterogeneity was analyzed and quantified from simulation results. Therefore, hypothesis H2 was corroborated.

The analysis provided the following results:

1. The spatial variability of the test area is dominated by inter-field variability. Intra-field variability does not contribute much to the overall variability. Therefore, a dataset of coarser resolution can be sufficient to infer LAI spatial variability. Therefore, in principle, a high resolution dataset is not necessarily required to infer the spatial variability in our test area. A dataset of field means is sufficient. However, since agricultural fields in the test area are irregular spatial structures, a field mean dataset cannot be acquired from the regular grids of medium resolution remote sensing.
2. LAI from the 150 m resolution simulation (*sim*) was expected to show lower LAI spatial variability than LAI form 5 m resolution remote sensing (*rs5m*) because of reduced variability of model input (soil properties, plant properties, crop management, and weather). However, since bare fields produce an LAI of exactly zero in the model whereas those from *rs5m* often show higher values in remote sensing data (measurement uncertainties, weeds, catch crops) SDs from *sim* and *rs5m* are similar. This emphasizes the importance of including plant growth that occurs on a field during absence of the main crop in simulations.
3. Each of the main crops shows a characteristic pattern of the temporal course of LAI relative frequency distributions (RFDs). Time series from the simulation help to understand the contribution of individual crops to the complex overall distributions at different points in time. RFDs from *sim* usually show narrower peaks and are less continuous than those from *rs5m*.
4. Describing RFDs using mean and SD implies that they are well-represented by a normal distribution. This does not apply to arable land with several crops that show multiple peaks. The RFDs may be described however as a superposition of the frequency distributions of the separate crops. Therefore, a reliable and validated model is more suitable to generate LAI fields with realistic frequency distributions than assuming a normal distribution.
5. The semivariograms used to analyze the spatial structure of LAI generally show irregular shapes. Quantification by theoretical semivariograms often required fitting of two nested semivariogram models, which demonstrates that there is more than a single effective length determining spatial structure. Ranges of the fitted models reveal short ranges less than 325 m that are attributed to field dimensions and long ranges above 1560 m that are supposedly connected to weather and soil properties. The long ranges were not determined in the simulation results. However, the noticeable increase of semivariance with increasing lags in the simulation results points towards spatial structures with even larger effective lengths that can only be detected on larger areas.
